# Status and determinants of saving behavior and intensity in pastoral and agro-pastoral communities of Afar regional state, Ethiopia

**DOI:** 10.1371/journal.pone.0281629

**Published:** 2023-02-16

**Authors:** Dagmawe Menelek Asfaw, Atinkugn Assefa Belete, Abibual Getachew Nigatu, Getnet Mamo Habtie

**Affiliations:** 1 Department of Economics, College of Business and Economics, Samara University, Samara, Ethiopia; 2 Department of Accounting and Finance, College of Business and Economics, Samara University, Samara, Ethiopia; 3 Department of Statistics, College of Natural and Computational Science, Samara University, Samara, Ethiopia; Universitat Jaume I, SPAIN

## Abstract

Saving is a crucial tool for enhancing the livelihoods of pastoral and agro-pastoral communities, but due to a number of factors, its status and intensity are still in their infancy. Because of this, the current state of saving practices, their causes, and the size of pastoral and agro-pastoral communities are all examined in this study. A multi-stage sampling process was used to determine the 600 typical selected households. In order to assess the data, a double hurdle model was used. From the descriptive analysis result, only 35% of pastoral and agro-pastoral groups were savers. In comparison to their counterparts, households who have access to credit, are financially literate, engage in non-farm activities, cultivate crops in addition to livestock husbandry, use informal financial institutions, are educated, and wealthier are more likely to be savers and eager to save a larger amount of property. Households with more livestock and who live far away from formal financial institutions, on the other hand, are less likely to be savers and save only a small fraction of their income. Male-headed families are more likely to participate in saving decisions, whereas female-headed households must save more than their male counterparts once they have opted to save. Instead of relying on ineffective monetary policy (changing interest rates), any concerned bodies should emphasize mixed farming practices, establish financial institutions nearby to improve saving habits, provide non-farm training, and empower women in order to close the gap between savers and non-savers and mobilize resources to save and invest. Furthermore, raise awareness of financial institutions’ products and services, as well as provide credit.

## Introduction

One of the most important goals for most economies is to achieve long-term growth. Various economists have long considered domestic savings to be one of the most important predictors of economic growth. According to classical economists like [1–3], saving is an important determinant of economic growth/ expansion. According to [4], any community must mobilize both local and foreign aid/savings to help them with essential investments that will encourage economic growth if they want to attain self-sustaining economic growth. Furthermore, according to [5], the endogenous growth hypothesis says that higher rates of savings and investment are necessary in an economy since they have a strong and positive association with GDP growth.

Countries with higher savings rates have had faster economic growth and capital accumulation than countries with lower rates. Capital accumulation provides a country with more opportunities for production and productivity by giving an additional revenue source [6–8] and also promote savings to mobilize cash in the most productive practice [9–11]. The advantages of saving at the household level include having a backup plan in case of an emergency, asset building, cash available for household investment, retirement planning. Savings can assist in the purchase of dwellings and housing, debt settlement, attaining dreams goals, long-term security, disaster protection, and the procurement of social services.

Even though, different national policies have been launched to improve the level and intensities of saving in Ethiopia, they have not achieved the expected outcomes. For example, [Ethiopia National Planning Commission [ENPC], 12], aim to increase and mobilize domestic savings to foster productive sector and job creation, however, the saving rate has been declining over time till 2021.

Based on the national bank of Ethiopia 2022 report, Ethiopia’s savings rate was declining from 24.1% of gross domestic product (GDP) in 2018 to 9.8% of GDP in 2021. The average Ethiopian household saves 875 birr each year in financial institutions, and this is insufficient to sustain the country’s economic growth and development [13]. This was less than the average of sub-Sahara countries’ saving rate (21%) and the least developing countries’ average (27%) in 2021 [World Bank, 14]. From the period 2015/16–2018/19, the saving-investment gap is on average 31% and 37% of GDP, respectively [National Bank of Ethiopia[NBE], 15]. Those savings rate decrements had an impact on shrinking both private and public investment. This was supported by a national bank report, 16 investment projects with a total investment capital of Birr 1.8 billion became operational in the first quarter of 2020/21. Both the number of investment projects and investment capital have slowed in comparison to previous years.

Investment is one component of GDP, therefore the decline of investment will have hurt the national economic growth as well as the livelihood of the nations [9]. This low saving rate is due to a variety of factors, including a lack of financial incentives for savers, limited access to financial institutions, low level of financial capabilities and awareness, financial exclusion [16], under-developed financial infrastructure, lack of documentation [17], lack of trust for formal financial institutions, poverty in money and social networks [18–20] and so on.

Saving is a versatile solution for an emergency like floods, drought, animal disease pandemics, war, and displacement. Saving helps as a contingency for emergencies like floods, drought, animal disease pandemics, war, and displacement. In addition, saving is a source of future capital formations and wealth; as initial capital for non-farm activities, in general, a means for pastoral and agro-pastoral community livelihood improvements. However, the status and intensity of saving is infant, for example [16] discovered that in Afar regional state only 31.255 of the households were included informal financial service and the remaining 68.75% of them were also excluded from it.

Different studies have been conducted to analyze the determinants of saving behaviors so far in Ethiopia as well as out of Ethiopia [6, 21–27] and so on, so far, no studies had been conducted to analyze the status and determinants of saving behaviors, and intensities in pastoral and ago-pastoral communities of the Afar regional state. This makes insufficient empirical evidence about the saving of the host communities; which again make it difficulty for policy maker to articulate a policy regarding rural financial resource utilizations. In addition, most of such studies were conducted only on the analysis of determinant factors for the decision to save/not to save by using binary models or OLS estimation method. This might not give full meaning of saving, rather we think also about the determinants of intensity (amount) of saving. This was because; variables that determined the decision to save might/might not be the determinants of intensity of saving and have not the same effect on both. As a result, this might leads to incorrect conclusion, policy recommendations, and provided inadequate information for policymakers. Therefore this study tried to analyze the status and determinants of saving behavior and intensity of Pastoral and ago-pastoral communities of the Afar regional state by using a double hurdle model.

## Methodology

### Description of the study area

Pastoralists and agro-pastoralists in Ethiopia are largely located in the country’s south and east, where they make up around 13% of the population and 63 percent of the land. The Afar Regional State, one of Ethiopia’s nine Federal states, covers 72,053 square kilometers and has a population of roughly 1.6 million people (estimated in 2012). With 22.2 people per square kilometer, it is a wide and sparsely inhabited territory in comparison to neighboring regions. The lowest point in Ethiopia is located in the north-eastern corner of the country. Between 39°34′ and 42°28′ East (longitude) and 8°49′ and 14°30′ North (latitude), the Afar area is located (latitude). It has a hot climate (25°C–48°C) and a flat environment with a height range of 116m below sea level to 1600m above sea level. Five zones, 32 wereda, 28 towns, and 401 rural and urban Kebelle (It is the lowest political administrative unit in the Ethiopia.) make up the Afar National Regional State. It shares borders with Eritrea to the north and Djibouti to the east, as well as Ethiopia’s Somali regional state to the south, Tigray to the north, Oromia to the south, and Amhara to the west [Central Statistical Agency [CSA], 28].

Rural areas account for 87 percent of the population, with pastoral and agro-pastoral livelihood systems. Women make up around 44% of the population, while men make up 57%. While the Afar regional state the population is predominantly Afar ethnicity, there is some ethnic diversity. The ethnic mix in 2008 was as follows: In terms of religious affiliation, 96 percent of the population is Muslim, while the rest is Christian. About 90% of the population relies on livestock production for a living, with irrigated agriculture limited to river basins and low-lying areas. In general, Afar communities engage in cattle raising not only for economic reasons but also for its social and cultural significance, as well as its connections to social values and kinship networks [CSA, 28].

### Sampling technique and sample size

The multistage sampling technique was applied to identify pastoral and agro-pastoral sampled households with proportional allocation. In the first stage, seven districts were selected randomly from each zone of Afar region, namely: Millie, Dallol, Afambo, Gewane, Yalo, Hadele Ele, and Asaita. In the second stage, select one pastoral and one agro-pastoral kebelle from each district. In the third stage, select 600 samples from each sampled kebelles by using systematic random sampling. The intended sample size was allotted to each sampled kebelles based on their population probability proportion (see Table 1).

**Table 1:** Sampling distribution and sample size.

Districts	Kebelles	Sampled households	Percentage proportions
Mille	Bekereda	50	8.26%
	01	49	8.15%
Dallol	Berih	32	5.34%
	Adokuwa	39	6.43%
Afambo	Humodeta	39	6.53%
	Alasabolo	40	6.70%
Gewane	Yegeli	45	7.56%
	01	41	6.74%
Yalo	Hadelella	40	6.62%
	Leleda	42	7.02%
Hadele Ele	Rekubdora	48	8.03%
	Mesgid	37	6.23%
Asaita	Beridaba	50	8.37%
	Kurudora	48	8.02%
**Total**		**600**	**100**%

Source: own computation, 2022

The planned sample sizes were determined by using [29] sample size determination formula as follows (Eq 1)

$$\text{n}=\frac{Z^{2}pq}{e^{2}}=\frac{{\left(1.96\right)}^{2}\left(0.5\right)\left(0.5\right)}{{\left(0.04\right)}^{2}}=600.25\approx 600$$ (1)

**Where**: n is the sample size; *z* is 1.96 to achieve 95% the level of confidence; p is 0.5 and q is 0.5; n is the sample size which is 600; *e* is the tolerant marginal error as defined as in 0.04, that is, 4% maximum discrepancy between the sample and the general population [30].

### Sources and methods of data collection

Primary data were employed to address the study’s intended goal. We used primary data, which came from 600 sampled households in four sampled kebelles. A structured and semi-structured questionnaire that addressed demographic, socioeconomic, institutional, and saving-related characteristics of the sampled households, was administered by a team of four trained enumerators consist of health extension workers for each sampled kebelles. The primary data was also collected from observation and key informant interviews with religious leaders, kebelle representatives, kebelle cabins, and other concerned bodies.

### Analytical framework

Two statistical techniques were used to assess the information gathered from the 600 sample respondents. First, descriptive statistical methods such as arithmetic means, standard deviations, percentages, and frequency were used to describe and assess the socioeconomic characteristics, institutional and saving characteristics of sample respondents in the study area; and inferential statistics method that was independent t-test and chi-squared test were applied to describe the variables used for analysis the statistically significant differences between savers and non-savers with regards to continuous variables and categorical variable respectively.

The second analysis was done using the econometric analysis approach to examine the determinants of the decision to save and the intensity of saving. The common approaches of modeling decision and intensity of saving are the Tobit [31, 32], Heckman, and double-hurdle models [22, 33]. However, in this study, we have used double-hurdle models to analyze the decision and intensity of saving in this study area.

The double hurdle model assumes producers are faced with two hurdles in any agricultural decision-making process [32, 34]. This model is a generalization of the Tobit, where the participation and quantity decisions are determined with two separate stochastic processes. In the first stage, applied the first hurdle (probit model) to analyze the saving decision of sampled households, and the second stage used a truncated regression model to analyze the extent(amount) of saving [22, 33].

The basic difference between Heckman and the double hurdle model is the assumption the Heckman that defines non-participants as a sample that will not participate under any circumstance (those zeros from non-participants are measured as unobserved or missed observation), even though, the double hurdle assumes that the decision not to save is a deliberate choice (thus the zeros from non-participants are takes as genuine zero that are corners solution in the utility-maximizing model) [35]. In the second stage of the double hurdle model, a subset of the data can pile up at a certain value without producing bias in estimating the determinants of the continuous dependent variable [31, 36–38]. The model is also flexible, which means there are no restrictions, on the components of explanatory variables in two estimation stages. As a result, the double hurdle model is a less restricted variant of the Heckman model that is best suited for samples collected using random probabilistic sampling processes, such as the one used in this study.

Finally, run a statistical test to choose between the Tobit and the Double-hurdle models for best fits the data using the likelihood ratio (LR) test [39].


$$\lambda =-2\left(LL_{T}-LL_{P}-LL_{TR}\right)$$ (2)

Where:-*LL*_*T*_, is log-likelihood values for the Tobit; *LL*_*P*_, is log-likelihood values for the Probit and *LL*_*TR*_ is log-likelihood values for the truncated. *λ* is an LR degree of freedom equal to the number of independent variables, statistic value with Chi-square distribution. Under the null hypothesis, the Tobit model is more appropriate than the double-hurdle model. As a result, the null hypothesis is rejected, implying that the double hurdle model is a superior fit for the data.

### Model specification

The double-hurdle model is designed to analyze the decision to save or not is made first (decision), followed by the decision on how much amount of money was going to save (outcome).

The decision to save is modeled as probit regression as follows (Eq 3):

$$d_{i}^{*}=X_{1i}\beta_{1}+\mu_{i},\;\;\;\;\;\;\mu_{i}\sim N\left(0,1\right)$$ (3)

$$d_{i}=\left\{\begin{array}{lll} 1 & if & d_{i}^{*}>0\\ 0 & if & d_{i}^{*}\leq 0 \end{array}\right.$$ (4)

The intensity (amount) of saving is modeled as a truncated regression as follow (Eq 5):

$$Y_{i}^{*}=X_{2i}\beta_{2}+\nu_{i},\;\;\;\;\;\;\nu_{i}\sim N\left(0,\sigma^{2}\right)$$ (5)

$$Y_{i}=\left\{\begin{array}{lll} Y_{i}^{*} & if & Y_{i}^{*}>0\;\;and\;d_{i}=1\\ 0 & if & Y_{i}^{*}\leq 0 \end{array}\right.$$ (6)

Where: *X*_1*i*_ and *X*_2*i*_ are vectors of explanatory variables that affect these two-stage decisions, respectively. μ_*i*_ *and ν*_*i*_ are uncorrelated error terms for both decisions, respectively.

*β*_1_ and *β*_2_ are the respective vectors of parameters. $d_{i}^{*}$ *is* Latent or unobserved decision to save (*if* $d_{i}^{*}=1$, *a* household is saver and $d_{i}^{*}=0$, households are non-saver), *d*_*i*_ *is* observed decision to save, *Y* is the observed amount of money to save and $Y_{i}^{*}$ is the latent or unobserved amount of money to save.

Assuming the error terms are independent and the stochastic specification in can be written as (Eq 7):

$$\left(\begin{array}{c} \mu_{i}\\ \nu_{i} \end{array}\right)\sim N\left[\left(\begin{array}{c} 0\\ 0 \end{array}\right),\;\left(\begin{array}{c} 1\\ 0 \end{array}\;\;\;\;\begin{array}{c} 0\\ \sigma^{2} \end{array}\right)\right]$$ (7)

The double-hurdle model with independent error terms can be estimated by the following log-likelihood function as follow (Eq 8):

$$LL=\sum ln\left[1-\phi \left(X_{1i}\beta_{1}\right)\Phi \left(\frac{X_{2i}\beta_{2}}{\sigma }\right)\right]+\sum ln\left[\phi \left(X_{1i}\beta_{1}\right)\frac{1}{\sigma }\Phi \left(\frac{Y_{i}-X_{2i}\beta_{2}}{\sigma }\right)\right]$$ (8)

Where: *Φ* (Greek capital letter phi) denotes the standard normal probability, *ϕ* (Greek small letter phi) is density functions; *X*_1*i*_ and *X*_2*i*_ represent independent variables for the probit model and the truncated model respectively; *β*_1_, *σ*, and *β*_2_ are parameters to be estimated for each case.

Therefore, the first hurdle (probit) model for determinants of the decision to save (*Save*_*i*_) was specified as follows (Eq 9):

$$\begin{array}{c} DSave_{i}=\beta_{0}+\beta_{1}edu_{i}+\beta_{2}credit_{i}+\beta_{3}finalit_{i}+\beta_{4}nonfarm_{i}+\beta_{5}finainc_{i}+\beta_{6}saftaid_{i}\\ +\beta_{7}cropp_{i}+\beta_{8}intr_{i}+\beta_{9}dstfins_{i}+\beta_{10}dep_{i}+\beta_{11}income_{i}+\beta_{12}sex_{i}+\beta_{13}ownliv_{i}+\;\vartheta_{i} \end{array}$$ (9)

The second hurdle model (truncated regression) for determinants of the amount to save (*Asave*_*i*_) was specified as follows (Eq 10)

$$\begin{array}{c} Asave_{i}=\alpha_{0}+\alpha_{1}edu_{i}+\alpha_{2}credit_{i}+\alpha_{3}finalit_{i}+\alpha_{4}nonfarm_{i}+\alpha_{5}finainc_{i}+\alpha_{6}saftaid_{i}\\ +\alpha_{7}cropp_{i}+\alpha_{8}intr_{i}+\alpha_{9}dstfins_{i}+\alpha_{10}dep_{i}+\alpha_{11}income_{i}+\alpha_{12}sex_{i}+\alpha_{13}ownliv_{i}+\varepsilon_{i} \end{array}$$ (10)

The parameter estimates (*α*) is specified that the signs of the partial effects of the independent variables on the estimated probability of the dependent variable (*Save*_*i*_). However, marginal effects of parameter estimates (*α*) help in assessing the effect of each independent variable on the dependent variable for the i^th^ sampled households [40].

### Definition of variables

The definitions of variables used in the double-hurdle to analyze the decision and intensity of saving are presented for the i^th^ sampled households as follows.

*The decision to save (DSave*_*i*_*)*: It is a dummy variable that indicates the decision of the household head to save/deposit properties in-kind/cash at formal financial institutions.

*The amount to save (ASave*_*i*_*)*: it is the total amount of money in Birr (it is the unit of currency of Ethiopia) deposited at formal financial institutions, and also it measures in a continuous form.

*Educational status of household head (edu*_*i*_*)*: it is years of schooling at formal education that the household head has completed till 2022 academic year, it is a continuous variable.

*Access of credit (credit*_*i*_*)*: is represented access of credit at 2022 fiscal year from formal financial institutions, that aimed to address the effect of credit on decisions and intensity of saving. It takes a value of “1” if the household head has access to credit and “0”, otherwise.

*Financial literacy (finalit*_*i*_*)*: it indicated that, the knowledge and skillsets regarding the financial sector products/services. It is a dummy variable, which was taken at a value of “1” if the household head has a knowhow and skill about financial sector products/services and “0”, otherwise.

*Participation in non-farm activities (nonfarm*_*i*_*)*: it shows that, the participation of household heads in non-farm activities like transport, distribution, marketing, retail, handicrafts, bakeries, mechanics, kiosks, and so on. If the household involves in such activities it takes “1” and “0” if the household doesn’t not.

*Financial inclusion (finainc)*: It refers to the provision of financial services to the general public at a reasonable cost. It measures as a binary variable, “1” if the household has inclusion in financial service at 2022 fiscal year, and “0” exclusion from financial service.

*Access of safety net aid (saftaid*_*i*_*)*: it is a dummy variable that households received/do not receive a safety net aid both in kind or cash at 2022 fiscal year. If a household gets safety net aid (both in-kind or money) “1”, if not “0”.

*Participation in crop production (cropp*_*i*_*)*: it is the participation of households in crop production like grain, fruit, biomass production, and dummy variable (“1” if households participate in crop production, “0” otherwise).

*Willingness to have deposit interest rate (intr*_*i*_*)*: it is the willingness of depositors not to have deposit interest rate. which is measured as a dummy variable, if the household is willing to have a deposit interest rate “1”, or if they are reluctant to have a deposit interest rate “0”.

*Distance to nearest financial institutions (dstfins*_*i*_*)*: It is a continuous variable and the distance from the farmer’s home to the nearest formal financial institution is measured in kilometers.

*Dependency ratio (dep*_*i*_*)*: It is a continuous variable, which is a demographic indicator that computed as, the number of dependents (those aged below 14 and above 64) divided by the total working-age population in a given households.

*Annum income (income*_*i*_*)*: It is aggregated amount of income in Birr that emanated from different sources of income. It is also measured as a continuous variable.

*Sex of household head (sex*_*i*_*)*: This is a discrete variable that takes a value of “1” if the household is male-headed and “0” female.

*Ownership of livestock (ownliv*_*i*_*)*: It is a continuous variable that refers to the total number of livestock in tropical livestock units (TLU) of the household members. TLU are livestock numbers converted to a common unit by using livestock conversion factor.

#### Ethical consideration and consent to participate

Ethical clearance was obtained from the College of Business And Economics, Samara University. Confidentiality of the information was secured by excluding respondents’ identifiers, such as names, from the data collection format. Finally, verbal informed consent was obtained from those who were in seven sampled districts and willing to participate in the study. Moreover, the results were recommended to be disseminated by the responsible bodies who were involved in the finance sectors.

## Results and discussion

### Descriptive analysis

The descriptive analysis of this study was conducted by descriptive statistics (Means, standard deviation, frequencies, and percentages) and inferential statistics (independent t-test and chi-squared test) to assess, compare, and check the relationship between dependent variables across independent variables. The descriptive comparison of categorical variables based on frequency counts and the chi-squared test is presented in Table 2.

**Table 2:** Cross-tabulation of saving decision with categorical independent & demographical variables.

Variables	Category	Decision to save		Total	Chi-square value
		Saver (n = 210 or 35%)	Non-saver (n = 390 or 65%)		
Non-farm	Participant	164(78%)	70(18%)	234(38.2%)	32.03***
	Not-Participant	46(22%)	320(82%)	366(61.8%)	
Credit access	Yes	179(85%)	47(12%)	225(37.55%)	22.28***
	No	15(15%)	343(88%)	375(62.45%)	
Marital status	Single	55(26.32%)	0(0.00%)	55(9.11%)	46.41***
	Married	155(73.68%)	328(84.06%)	483(80.47%)	
	Divorced	0(0.00%)	42(10.76%)	42(7.03%)	
	Windowed	0(0.00%)	20(5.18%)	20(3.39%)	
Crop production	Participant	144(69%)	55(14%)	200(33.25%)	52.76**
	Not-Participant	65(31%)	335(86%)	400(66.75%)	
Sex	Male	163(78%)	187(48%)	351(58.5%)	25.17***
	Female	47(22%)	203(52%)	249(41.5%)	
Safety net aid	Yes	122(58%)	183(47%)	305(50.85%)	36.28***
	No	88(42%)	206(63%)	295(49.15%)	
Financial Inclusion	Yes	181(86%)	42(11%)	223(37.25%)	31.37***
	No	30(14%)	347(89%)	377(62.75%)	
Financial literacy	Yes	183(87%)	70(18%)	253(42.15%)	7.12**
	No	27(13%)	319(82%)	347(57.85%)	
Interest rate	Reluctant	183(87%)	374(96%)	557(92.85%)	19.09***
	Willing	27(13%)	15(4%)	42(7.15%)	

***,**, and * stand for significance at p<0.001, P<0.05, and P<0.1, respectively, Source: Own survey, 2022

According to this study, from the total sampled households, 210 (35%) were savers in the formal financial institution, while, 390 (65%) of them are non-savers. The results under Table 2 reveal that among savers, 78% participated in non-farm activities, which was greater, compared to 18% among non-saver. However, from the total sample households, 61.8% did not participate in non-farm activities. The households were eager to save money because of an increase in non-farm income. Therefore, chi-square results suggest a positive relationship between non-farm participation and the decision to save. In addition, Table 2 found that only some proportion of the total sample (37.55%) had access to credit. However, among the participant subsample, 85% were exposed to credit compared to 12% among non-participants. Credit availability increases the likelihood of saving money and strengthens one’s ability to make investments. This suggests a positive association between access to credit and the decision to save.

The analysis revealed that 84.06% of savers were married compared to 15.94% of others (single, divorced, and windowed), implying a positive association between marital status (married) and the decision to save. From the saver sub-sampled households, crop producers (69%) were higher than their other counterparts (31%), even though 66.75% of the total sample was pure pastoralist. Mixed farming could be a source for additional income and create opportunities for mobilized income to save (see Table 2).

The results indicate that male households were dominant, making up 58.5% of the total sample. Even though out of the total sampled households, 41.5% were female, and only 22% were savers compared to the 78% within the male population. This might be because the female household head had weak financial capabilities and was less likely to participate in saving. Because, most of the time women participated in less/unpaid activities, this in turn lead to women having weak financial capabilities. Among the participants, 58% had access of safety net aid compared to the 47% among non-participants. However, 49% of the sample population had no access to safety net aid (see Table 2).

Households with financial know-how and information will have enthusiastic to be financial inclusion and mobilizing their money to save in financial institutions. Among the savers’ sample households, 86% and 87% of them were financially included and financially literate, respectively. Whereas, 89% and 82% of non-savers are financial exclusion and financially illiterate, respectively. Therefore, the Chi-square revealed that there is a positive association between financial literacy and inclusion in saving decisions. More than 80% of this study area residents are Muslims, and therefore interest rate charge is considered as strictly forbidden (*haram)*. In such away, only 7.1% of sampled households have the willingness to accept deposit interest rate, and 92.8% of them are reluctant to deposit interest rate (see Table 2).

Education enhances the ability, knowledge, and skill of how to manage and mobilized resources including money. Based on Table 3, on average, the educational level of the household head is 2.7 and 0.52 for savers and non-savers households, respectively. Therefore, being educated is a means to being a saver for sampled households, this is justified by the t-test, and household education is statistically and positively related to the decision to save.

**Table 3:** Decision to save with continuous independent variables.

Variables	Decision to save				t-value	Total	
	Saver (n = 210 or 35%)		Non-Saver (n = 390 or 65%)				
	Mean	Sta. Dev	Mean	Sta. Dev.		Mean	Sta.Dev.
Education	2.79	3.887	0.52	0.023	-8.28***	1.82	3.410
Dependency ratio	0.59	0.387	0.57	0.564	-0.19	0.58	0.244
Annum income	46,780	752	27,568	569	-32.40***	32,568	652
Distance to financial institution	7.01	1.229	21.23	7.691	-1.18*	14.61	10.708
Livestock ownership(TLU)	58.23	19.972	33.52	14.427	-16.27***	43.25	17.270

***,**, and * stand for significance at p<0.001, P<0.05, and P<0.1, respectively

Source: Own survey, 2022

The average annum income is 46,780 and 27,568 birr for savers and non-saver, respectively. The t-test justified that, annual income is statistically related to the decision to save. The higher the income level the lower the marginal propensity to consume and the higher the marginal propensity to save. The households with livestock have a chance to get additional income and induced those households to save money. This is also justified by the t-test that the average amount of livestock owned by savers (58.23) is statistically and significantly higher than non-saver (33.52) (see Table 3).

The formal financial institution was available at 7.01 and 21.23 kilometers away from the sampled households dwelling on average, for participants and non-participants, respectively. This result showed that distance to a financial institution has a negative and statistically significant effect on the decision to save based t-test (see Table 3).

### Econometric results

Before proceeding to inferential analysis, all the hypothesized explanatory variables were checked:- for multicollinearity problems, using variance inflation factor (VIF) and contingency coefficients (CC) for continuous and categorical variables, respectively [40]; Heteroskedasticity using *Breusch-Pagan* test [39]; Omitted variable test using Ramsey test and normality test using a kernel density plot [41]. Therefore the test results found that there was no strong collinearity between explanatory variables, the variance of the error term was constant conditional on the chosen value of the explanatory variables, no omitted variables in the model, and the error term is normally distributed with its mean and variance. The LR test (for choosing between the double-hurdle model and the Tobit model) rejected. Tobit model specification and chose Double-hurdle model. This is an indication of the existence of two separate decision-making stages in which individuals make independent decisions regarding the decision to save and the amount to save.

### Determinants of the decision to save

To determine the factors influencing the decision to save using a Probit model, which was estimated in the first step of the double hurdle model equation.

The majority of the communities in this study area are pastoralists and herding livestock is their permanent source of income. Therefore, as the herd size of livestock increases, they are obliged to find forage and water for their livestock, and live far away from main cities and financial institutions. According to Table 4, the likelihood of being saved reduces by 33% when livestock ownership increases by one TLU. This finding is similar to a studies of [23, 33]. The interest rate was an insignificant determinant for both probability and intensity of saving. As stated before more than 80% of the study area residents were Muslim, and interest rate charge was considered forbidden (*haram*). Therefore, any change of interest rate (monetary policy) in this study area was inappropriate.

**Table 4:** Estimates from double-hurdle and Tobit models.

Variables	Double hurdle model					Tobit Model	
	Probit (first hurdle)			Truncated (second hurdle)			
	Coef.	Std.Err.	ME	Coef.	Std.Err.	Coef.	Std.Err
Livestock ownership	-0.97	-0.092	-0.33***	0.25	0.245	0.18*	0.097
Interest rate	0.72	0.195	0.24	0.09	0.101	0.15	0.147
Credit	0.48	0.079	0.16**	0.17	0.129	0.19***	0.073
Financial literacy	1.41	0.126	0.46***	0.28***	0.080	0.02**	0.010
Financial ins^n^ Distance	-0.46	-0.071	-0.14**	-0.17**	-0.084	-0.03	0.058
Non-farm activities	0.98	0.227	0.35	0.22***	0.076	0.36**	0.177
Financial inclusion	0.51	0.119	0.16	0.35***	0.122	0.43	0.422
Dependency ratio	-0.34	-0.109	-0.121	-0.12	-0.012	-3.05**	-1.473
Education	0.67	0.101	0.235**	0.41	0.301	0.24	0.229
Safety net aid	-0.52	-0.184	-0.180	0.85	0.552	0.75	0.843
Crop production	0.05	0.019	0.019	0.41***	0.138	0.41	0.719
Annual income	0.06	0.02	0.021**	-0.34	-0.347	0.57	0.640
Sex	0.50	0.079	0.17**	-0.31***	-0.088	-0.37***	-0.385
Constant	-0.97	-0.092		-0.401	0.245	12.01	0.097
Sigma	0.83(0.190)					8.37(0.821)	
Log-Likelihood	-739.2					-923	
Prob > chi2	0.0000					0.0000	
Pseudo R2	0.42					0.286	
Likelihood ratio test: Tobit Vs Double-hurdle λ = 97							

ME denotes the marginal effect of the explanatory variables

***, **and *indicates statistically significant at p < 0.001, p < 0.05, and p < 0.1, respectively

Credit is important for mitigating cash-constrained and a source of startup capital for non-farm activities for pastoral communities. This might be a reason to generate an additional income that would improve the household’s eagerness to save in formal financial institutions [26]. As reported in Table 4, the likelihood of households with access to credit being savers is 16% compared to the counterparts with no access to credit. This finding was consistent with the result [21, 24, 42, 43].

Financially literate households can grasp and differentiate financial possibilities, feel at comfort discussing personal finance concerns, make decisions that protect against future insecurities, can understand the importance of various financial institution services, eager to invest and save their financial resource [16, 44–46]. The estimated result show that, the probability of saving is 46% for households who are financially literate compared to the illiterate, ceteris paribus (see Table 4). The result was in line with the work of (Gaisina & Kaidarova, 2017; Mahdzan & Tabiana, 2013; Egwu & Nwibo, 2014; Sanderson et al., 2018; [47].

The results in Table 4 show that, as financial institutions are far away from households dwelling by a kilometer, the probability of being a saver is decreased by 14%. This is because, as financial institutions are far away, the households are excluded from financial services (like credit, load, and saving), incur higher transaction costs, difficult to get updated financial information and service, and finally less eager to save. This result is consistent with the results [25, 27, 48].

Educated households have the readiness to accept new ideas and innovations, develop the know-how to diversify their source of income, and how mobilize this resource. Education is also the main determinant of higher earnings and this makes the households to be a saver. As year of schooling increases by 1 grade, the probability of households being savers will be induced by 23%. This is in line with the findings of [22, 33, 43, 49]; whereas, contrast with [24].

The coefficient of annual income was statistically significant and positively related to the decision to save. As the household’s income increases by a birr, the likelihood of the household being a saver increases by 2.1% (see Table 4). Saving is a portion of income that is a residual after consumption deducted from income, therefore any change in income is attributed to the change in saving. And also as income increase beyond a certain level, the marginal propensity to consume is going to be declined, and the marginal propensity to save starts to improve. As a household head becomes wealthier and wealthier, he /she want to secure their properties in safe place (formal financial institution) and start to save in such financial institutions. This result resembles the studies like [23, 25, 27, 50].

According to Table 4, a male household head has a 17% higher chance of becoming a saver than a female counterpart. This may be due to, females are most of the time dealing with household self-sufficient and home works rather than income generating activities, work for lower wages, have a less productive resource available than their male counterparts, and gender bias against women and bear the burden of household chores [31]. Male household heads have more exposure and access to information on financial services and actively participated in high-income-generating jobs. A similar result was also reported by [23, 24, 51].

### Determinants of intensity (amount) of saving

To determine the factors that affect the intensity of saving truncated model was employed in the second step of the double hurdle model. The results presented in Table 4 found that, when the households are being financially literate, the amount of saving increases by 28 Birr compared to the illiterate one. This was because, financially literate households were settling on sound monetary choices about how much to invest, and how much to save for future investment and contingency purposes. This finding is similar to: [27, 45, 46].

As the distance of financial institutions from household dwellings increases by a kilometer, the amount of savings is declining by 17% (See Table 4). When formal financial institutions were found far away from the household’s residence, there will be a higher risk associated with cash movement, difficulty to access the benefit and updated financial service information, and higher transaction costs. This result is consistent with the results: [13, 22, 33, 43, 52].

The double hurdle model revealed that, as the households participate in non-farm activities, their amount of saving at formal financial institutions is enhanced by 22%, compared to the non-participants. This is because, when households have participated in non-farm activities in addition to animal husbandry like petty trading, hairdressing, sewing, marketing, retail, handicrafts, bakeries, mechanics, kiosks, and so on, will lead to accumulate more income and be eager to save more than those who are not participating. This result is similar with [22, 42, 53].

A house with access to financial institutions and inclusion in financial services would excited them to save more. In addition, a household’s inclusion in financial services will have a probability to have financial know-how about mobilizing resources into investment and saving. Therefore, as a household included in the financial sector, the amount of saving increases by 35%, compared to the financial exclusion households. This result was advocated by [13, 54].

Result under Table 4 reported that, in households who are participating in crop production in addition to livestock husbandry, the amount of saving is improved by 41%, relative to the non-participants. When a household practices mixed farming, they accumulate more capital are able to diversify their income and nutrition source, and benefit from byproducts and productivity growth, this will leads to saving more. This result is similar with, [6, 51, 55].

For male-headed households, the amount of savings is decreases by 31%, compared to the female counterparts (See Table 4). The probit model found that the probabilities of male-headed households are higher than the female ones. However, after they do so, they tend to save less compared to a woman. In contrast, women are less likely to save but once they do, they tend to save more than males. This is due to, females are most of the time working on family self-sufficiency and child care, and they do not spend the remaining income on other unnecessary and unproductive activities but rather save it. However male-headed households spend their residual income for their enjoyment and recreation rather than saving it. This finding similar with: [21, 23, 33].

## Conclusion and recommendation

The saving status of Afar pastoral and agro-pastoral communities is still infant and underdeveloped. In the descriptive analysis, only 35% of pastoral and agro-pastoral communities were savers and the remaining 65% of them were not saver at formal financial institutions. The double hurdle model revealed that households who: get access to credit, are financially literate, involved in non-farm activities, cultivate crops in addition to livestock husbandry, included in informal financial institutions, are educated, and are wealthier are more likely to be a savers compared to the counterparts. However, a household who had more livestock and lives far away from formal financial institutions is less probable to be a saver and save a small portion of income compared to others. Male-headed households have more probability to participate in saving decisions compared to female counterparts, whereas once female-headed households are decided to save, they have to save more amount than their male counterparts.

To narrow the gap between savers and non-saver, mobilize resources to save and invest, and improved the amount of savings, this study suggested that: the households should practicing mixed farming; the government should also empowering women to participate in formal financial institutions, creating awareness about financial institutions’ product and service, offering credit. Provided a business related training about petty trading, hairdressing, sewing, marketing, retail, handicrafts, bakeries, mechanics, kiosks, and so on. The financial institution should be established nearby the residents of the household to improve their saving habits rather than use ineffective monetary policy (change interest rate). Further research is suggest on analyzing the decision and intensity of saving habits in informal and formal financial institutions and also in non-financial institutions, based on the finding of this study.

## Supporting information

S1 Data (DTA)
